# Mortality Prediction in Patients Undergoing Non-Invasive Ventilation in Intermediate Care

**DOI:** 10.1371/journal.pone.0139702

**Published:** 2015-10-05

**Authors:** Diego Martinez-Urbistondo, Félix Alegre, Francisco Carmona-Torre, Ana Huerta, Nerea Fernandez-Ros, Manuel Fortún Landecho, Alberto García-Mouriz, Jorge M. Núñez-Córdoba, Nicolás García, Jorge Quiroga, Juan Felipe Lucena

**Affiliations:** 1 Clínica Universidad de Navarra, Department of Internal Medicine, Division of Intermediate Care and Hospitalists Unit, Pamplona, Spain; 2 Instituto de Investigación Sanitaria de Navarra (IdiSNA), Pamplona, Spain; 3 Clínica Universidad de Navarra, Information Technology, Pamplona, Spain; 4 Clínica Universidad de Navarra, Division of Biostatistics, Research Support Service, Central Clinical Trials Unit, Pamplona, Spain; 5 Department of Preventive Medicine and Public Health, Medical School, Universidad de Navarra, Pamplona, Spain; 6 Epidemiology and Public Health Area, Instituto de Investigación Sanitaria de Navarra (IdiSNA), Pamplona, Spain; 7 Centro de Investigación Biomédica en Red de Enfermedades Hepáticas y Digestivas (CIBEREHD), Pamplona, Spain; Lee Kong Chian School of Medicine, SINGAPORE

## Abstract

**Background:**

Intermediate Care Units (ImCU) have become an alternative scenario to perform Non-Invasive Ventilation (NIV). The limited number of prognostic studies in this population support the need of mortality prediction evaluation in this context.

**Objective:**

The objective of this study is to analyze the performance of Simplified Acute Physiology Score (SAPS) II and 3 in patients undergoing NIV in an ImCU. Additionally, we searched for new variables that could be useful to customize these scores, in order to improve mortality prediction.

**Design:**

Cohort study with prospectively collected data from all patients admitted to a single center ImCU who received NIV. The SAPS II and 3 scores with their respective predicted mortality rates were calculated. Discrimination and calibration were evaluated by calculating the area under the receiver operating characteristic curve (AUC) and with the Hosmer-Lemeshow goodness of fit test for the models, respectively. Binary logistic regression was used to identify new variables to customize the scores for mortality prediction in this setting.

**Patients:**

The study included 241 patients consecutively admitted to an ImCU staffed by hospitalists from April 2006 to December 2013.

**Key Results:**

The observed in-hospital mortality was 32.4% resulting in a Standardized Mortality Ratio (SMR) of 1.35 for SAPS II and 0.68 for SAPS 3. Mortality discrimination based on the AUC was 0.73 for SAPS II and 0.69 for SAPS 3. Customized models including immunosuppression, chronic obstructive pulmonary disease (COPD), acute pulmonary edema (APE), lactic acid, pCO2 and haemoglobin levels showed better discrimination than old scores with similar calibration power.

**Conclusions:**

These results suggest that SAPS II and 3 should be customized with additional patient-risk factors to improve mortality prediction in patients undergoing NIV in intermediate care.

## Introduction

Non-Invasive Ventilation (NIV) reduces in-hospital mortality in selected patients with acute respiratory failure (ARF) [1–4]. Currently, indications and success of this ventilation mode depend on ARF etiology, the evolution in the first hours of the PaO2/FiO2 ratio, pH, pCO2 and the respiratory rate [5–7]. For this reason, initiation of NIV in ARF should be done under continuous surveillance [5]. In this context, Intermediate Care Units (ImCU) may offer a suitable alternative to the intensive care unit (ICU), due to the availability of non-invasive monitoring and specialized staff and nursing dedication [8].

Statistically significant differences in Simplified Acute Physiology Score (SAPS) II and Acute Physiology and Chronic Health Evaluation (APACHE) II scores have been found between survivors and non-survivors who underwent NIV due to acute lung injury (ALI) [9], acute respiratory distress syndrome (ARDS) [10], COPD exacerbation [11], acute pulmonary edema (APE) [12], interstitial lung disease [13] and acute myasthenia gravis [14]. Regarding the performance of SAPS 3, a revised version of SAPS II, which includes more comorbidities and physiological values within the first hour of admission [15], the information is even more limited. Metnitz et al., [16] described a cohort of 157 patients who underwent Pressure Support Ventilation (PSV) from a multicenter multinational study of the SAPS 3 data base. The study was descriptive, without information regarding the calibration and discrimination power of the score.

In this context, conventional scores show high dependence on case-mix for accurate prediction [17–19] and do not include important respiratory variables, such as ARF etiology, hemoglobin and pCO2 levels [20–24]. Therefore, the objectives of this study were: (i) to describe the outcomes of NIV population in an ImCU, (ii) to evaluate the performance of SAPS II and 3 in mortality prediction of patients undergoing NIV in this area and (iii) to customize the scores with new variables in order to improve their performance.

## Materials and Methods

The study was performed at the Clínica Universidad de Navarra, an academic medical center in Pamplona, Spain. Our ImCU is a 9-bed multi-purpose area staffed by hospitalists and shared with coronary and stroke units while being independent from the mixed ICU. Each bed is equipped with continuous telemetry, pulse-oxymetry, noninvasive arterial blood pressure, central venous pressure monitoring, and non-invasive pressure support ventilation with BiPAP. The signals are relayed to a central monitoring station and the nurse-patient ratio is 1:3. Demographics, past medical history, reasons for admission, physiological parameters at the time of admission and during the first 24 hours of ImCU stay, laboratory variables and survival to hospital discharge were prospectively recorded by the authors. Admission and discharge criteria of the ImCU were set according to previous guidelines defined by The American College of Critical Care Medicine [8].

Every consecutive patient undergoing NIV from April 2006 to December 2013 was evaluated and included in the study. Exclusion criteria from ImCU admission were: age less than 18 years, severe respiratory failure at imminent risk of intubation, status epilepticus and catastrophic brain illness. ImCU readmissions were also excluded from the final analysis. In-hospital mortality was the end point of the study.

Indication of NIV was established according to previously published guidelines [5]. Patients were divided in four groups of ARF etiology: COPD exacerbation, acute pulmonary edema, hypoventilation due to neuromuscular disease and hypoxemic ARF. NIV failure was defined as the discharge of patients to the ICU to be intubated or the in-hospital death of subjects due to ARF when do-not resuscitate (DNR) orders were set.

### Ethical Standards

The study protocol was approved by the Institutional Review Board (IRB) at the Clínica Universidad de Navarra (ref. 129/2010). The IRB waived the need for informed consent, because it did not interfere with decisions related to patients care. The study has been performed in accordance with the ethical of the 1964 Declaration of Helsinki and its later amendments.

### Statistical Analysis

Data were prospectively collected after patient admission, into a computer database by the authors and based on the electronic medical record system of the Clínica Universidad de Navarra. Continuous variables were reported as mean and standard deviation, and as median and interquartile range (IQR) when appropriate. These variables were analyzed by t-Student, ANOVA, U-Mann Whitney and Kruskal Wallis tests. Categorical variables were summarized as absolute frequencies and percentages, and analyzed by chi-square test. Performance of the prognostic scores was assessed by standard procedures to measure discrimination and calibration. Discrimination was evaluated by calculating the area under receiver operating characteristic (ROC) curve (AUC) [25] and calibration was assessed using the Hosmer-Lemeshow goodness-of-fit test, in which a high p value (> 0.05) was interpreted as a good fit for the models [26, 27]. In the rest of the analyses, statistically significant results were considered when p <0.05. Standardized mortality ratios (SMR) (ratio of observed to expected deaths) were calculated for each model and ARF etiology subgroup. Customized models were developed using binary logistic regression. The variables included in the model were: hemoglobin, pCO2 and lactic acid levels, SAPS II score, COPD diagnosis and APE, previously described in NIV mortality prediction [5,21–24]. Immunosuppression, DNR orders and the SAPS 3 score, were also included in the analysis, due to their potential clinical application in predicting NIV mortality.

Statistical analyses was performed, using SPSS for Windows, version 20.0 (SPSS Inc, Chicago, IL).

### Model Validation

The customized models were validated internally by bootstrapping. The bootstrap method randomly draws multiple samples with replacement from the original cohort. The model is developed again in each bootstrap sample yielding a different AUC (c-statistic) to each bootstrap model [28]. An average of these c-indexes was calculated. The bootstrap procedure was performed with 1000 draws with replacement in the study cohort.

## Results

From April 2006 to December 2013, a total of 241 patients were non-invasively ventilated in the ImCU of our hospital and constituted the study group. Demographic characteristics, co-morbidities, location prior to admission, discharge location, variables at admission and length of hospital stay are described in Table 1. The population had a mean age of 68 years, and a female percentage of 37.3%. Most frequent co-morbidities were hypertension (55.6%), immunosuppression (56.2%) and COPD (29.9%). The general ward was the principal location prior to admission (63.5%) followed by the emergency room (23.3%) and the ICU (9.1%). Most patients were discharged to the general ward (67.6%) or to the ICU (19.1%).

**Table 1:** Patient characteristics.

**n**	241
**Demographic characteristics**	
Female sex, n (%)	90 (37.3)
Age in years	68 (13)
**Co-morbidities**	
Hypertension, n (%)	134 (55.6)
Diabetes mellitus, n (%)	63 (26.1)
Coronary artery disease, n (%)	46 (19.1)
COPD diagnosis, n (%)	72 (29.9)
Metastatic cancer, n (%)	59 (24.5)
Immunosuppression*, n (%)	139 (56.2)
**Location prior to admission**	
ICU, n (%)	22 (9.1)
Emergency room, n (%)	56 (23.2)
General ward, n (%)	153 (63.5)
Operating room, n (%)	1 (0.4)
Other hospital, n (%)	9 (3.7)
**Discharge location**	
General Ward, n (%)	163 (67.6)
ICU, n (%)	46 (19.1)
Other hospital, n (%)	6 (2.5)
Death at ImCU, n (%)	24 (10.0)
Home, n (%)	2 (0.8)
**Variables at admission**	
Heart rate in bpm	98.0 (20.8)
Systolic BP in mmHg	120 (26.1)
C-RP in mg/dl	13.4 (11.1)
PaO2/FiO2 ratio < 200 mmHg, n (%)	26 (10.8)
PCO2 in mmHg	45.4 (15.6)
pH	7.39 (0.09)
Lactic acid (mg/dl)	2.0 (1.5)
Hemoglobin (gr/dl)	11.1 (2.1)
**ImCU stay (days)**	5.83 (5.50)

Values are expressed as mean (standard deviation), unless otherwise stated.

SD: Standard deviation; COPD: chronic obstructive pulmonary disease; ICU: Intensive care unit; ImCU: Intermediate care unit; BP: Blood pressure; C-RP: C-reactive protein

*Immunosuppression as described in SAPS 3: Use of chemotherapy, radiotherapy, immunosuppressive drugs or corticosteroids in the last 6 months

Reasons for NIV application in the ImCU were COPD exacerbation (18.25%), hypoxemic acute respiratory failure (56.85%), acute pulmonary edema (17.01%) and neuromuscular disease (7.89%), as shown in Table 2. The expected mortality was 24.02+/-17.91% for SAPS II (median: 18% IQR: 11–34%) and 45.75+/-22.68% for SAPS 3 (median: 44% IQR: 28–63%), while the observed in-hospital mortality was of 32.4% (78/241 patients), resulting in a SMR of 1.35 for SAPS II and of 0.69 for SAPS 3. Mortality location distribution was: 19/78 at the ImCU (24.3%), 24/78 at ICU (30.7%) and 35/78 at the general ward (45%).

**Table 2:** Indications for NIV and mortality.

	Total	COPD exacerbation	Hypoxemic ARF*	Pulmonary Edema	Neuromuscular disease	p †
**n**	241	44	137	41	19	
**DNR orders, n (%)**	72 (29.9)	9 (20.5)	50 (36.5)	9 (22.0)	4 (21.1)	<0.01
**NIV failure, n (%)**	85 (35.3)	6 (13.6)	67 (48.9)	11 (26.8)	1 (5.3)	<0.01
**Mortality, n (%)**	78 (32.4)	9 (20.5)	58 (42.3)	9 (22.0)	2 (10.5)	<0.01
**SAPS II Expected mortality %** **	18 (11–34)	15 (11–23)	20 (12–35)	23 (12–38)	15 (6–35)	0.05
**SAPS 3 Expected mortality %** **	44 (28–63)	37 (26–45)	50 (30–67)	58 (51–64)	24 (10–44)	<0.01
**SMR SAPS II**	1.35	1.10	1.63	0.85	0.55	
**SMR SAPS 3**	0.69	0.54	0.86	0.43	0.21	

Values are expressed as mean (standard deviation), unless otherwise stated.

DNR: Do not resuscitate; SAPS Simplified acute physiological score; SMR: Standardized mortality ratio; COPD: Chronic obstructive pulmonary disease; ARF: Acute respiratory failure.

*Hypoxemic ARF group includes lower tract respiratory infection (n120), ALI/ARDS (n15) and Pulmonary Embolism (n2)

**Data are expressed as median and IQR due to small subgroup size.

^†^p of comparison between subgroups was calculated with chi-square and Kruskal Wallis tests.

Mortality prediction based on SAPS II showed conflicting results, considering the NIV indication. A mismatch was observed between the SMR of the hypoxemic respiratory failure and the neuromuscular disease groups (1.63 vs 0.55, respectively). However, the prediction power was acceptable for the groups of COPD (1.10) and APE (0.85). Interestingly, SAPS 3 tended to overestimate mortality in all the groups according to SMR, being more accurate for hypoxemic ARF (0.86) (Table 2).

Performance of SAPS II and 3 is described in Table 3. Discrimination according to AUC was of 0.73 (CI 95% 0.69–0.80) for SAPS II and 0.69 (CI 95% 0.63–0.77) for SAPS 3. Results from the Hosmer-Lemeshow goodness-of-fit tests showed a good calibration for both scores: (χ^2^ = 7.35, p = 0.49) for SAPS II and (χ^2^ = 4.45, p = 0.81) for SAPS 3.

**Table 3:** Performance of the scores.

	Mean score (SD)	AUC (IC 95%)	Hosmer-Lemeshow Test, χ^2^ (p)	SMR (O/E ratio)
**SAPS II**	37.56 (11.47)	0.73 (0.69–0.80)	7.35 (0.49)	1.35
**SAPS 3**	64.85 (14.03)	0.69 (0.63–0.77)	4.45 (0.81)	0.69
**SAPS II-CNIV**	36.67 (23.49)	0.84 (0.78–0.89)	5.62 (0.69)	NA
**SAPS 3-CNIV**	49.21 (39.85)	0.79 (0.73–0.85)	8.41 (0.39)	NA

SMR: Standardized mortality ratio. O/E: Observed/expected. NA: Not applicable

Univariate analysis was done for mortality prediction, including SAPS II, SAPS 3 and other important variables in the ARF context. Statistically significant differences were found for NIV indications, immunosuppression, patients with do-not-resuscitate orders (DNR), previous diagnosis of COPD, pCO2 (mmHg), lactic acid (mg/dl), hemoglobin (g/dl), SAPS II and SAPS 3 (p<0.05 for all) (Table 4). Other variables such as hypertension, coronary artery disease and white blood cell count were found to be not statistically significant in this analysis.

**Table 4:** Univariate analysis of new variables for NIV mortality prediction.

Variables	Death	Discharged	p^†^
**SAPS II**	44.00 (12.62)	34.48 (9.47)	<0.01
**SAPS 3**	70.87 (14.31)	61.97 (12.98)	<0.01
**NIV indication, n** *	9/58/9/2	35/79/32/17	<0.01
**Immunosuppression, n (%)**	72 (54.10)	67 (45.90)	<0.01
**DNR, n (%)**	37 (51.40)	35 (48.60)	<0.01
**Previous diagnosis of COPD, n (%)**	13 (18.10)	59 (81.90)	<0.01
**pCO2**	39.66 (14.64)	48.18 (15.33)	<0.01
**Lactic acid**	2.60 (1.92)	1.67 (1.19)	<0.01
**Hemoglobin (g/dl)**	10.60 (1.92)	11.33 (2.15)	<0.01

Values are expressed as mean (standard deviation), unless otherwise stated.

*COPD/hypoxemic ARF/APE/Neuromuscular disease

^†^p of comparison between subgroups was calculated with t-Student and chi square tests

All these variables were converted to dichotomous after sensitivity analysis and included together with SAPS II and 3 in independent logistic regression models. Then, two customized models (SAPS II-CNIV and SAPS 3-CNIV) were developed from logistic regression results (Tables 5 and 6).

**Table 5:** Logistic regression and score customization for SAPS II.

Variables	OR (CI 95%)	p	Score value
SAPS II (per point)	1.08 (1.04–1.12)	<0.01	+1
Lactic acid > 2 mg/dl	2.08 (1,03–4.20)	0.04	+10
Immunosuppression*	3.19 (1.56–6.50)	0.01	+15
COPD previous diagnosis	0.35 (0.16–0.79)	0.02	-14
Acute pulmonary edema	0.35 (0.14–0.90)	0.03	-14
pCO2> 55 mmHg	0.46 (0.19–1.12)	0.09	-10
Hemoglobin (> 10.7 g/dl)	0.47 (0.24–0.92)	0.03	-10

*Immunosuppression as described in SAPS 3: Use of chemotherapy, radiotherapy, immunosuppressive drugs or corticosteroids in the last 6 months

**Table 6:** Logistic regression and score customization for SAPS 3.

Variables	OR (CI 95%)	p	Score value
SAPS 3 (per point)	1.03 (1.01–1.06)	0.02	+1
Lactic acid > 2 mg/dl	2.39 (1.22–4.65)	0.01	+28
DNR orders	2.24 (1.12–4.46)	0.02	+25
COPD at admission	0.36 (0.17–0.78)	0.01	- 32
Acute pulmonary edema	0.39 (0.16–0.94)	0.04	- 26
pCO2> 55 mmHg	0.48 (0.21–1.14)	0.09	- 23
Hemoglobin (> 10.7 g/dl)	0.45 (0.23–0.86)	0.02	- 26

In the SAPS II-CNIV model, immunosuppression and lactic acid over 2 mg/dl were found to be risk factors, while previous diagnosis of COPD, acute pulmonary edema and haemoglobin levels over 10.7 g/dl were found to be protective factors (p<0.05). Carbon dioxide pressure (pCO2) > 55 mmHg was protective and near to statistical significance (p = 0.09). Interestingly, DNR orders were not statistically significant in this model (p = 0.20), probably due to a colinearity phenomenon (comorbidities, immunosuppression and metastatic cancer). Each variable was given a prognostic value to be included in the customized score, which was established as the weight of each variable in mortality prediction in comparison to a SAPS II single point (Table 5).

The SAPS 3-CNIV model showed statistical significance of DNR orders and lactic acid > 2 mg/dl as risk factors and previous diagnosis of COPD, APE and haemoglobin levels > 10.7 g/dl as protective factors (p<0.05). Meanwhile, pCO2 > 55 mmHg was a protective factor near to statistical significance (p = 0.09). Immunosuppression was not included in the analysis to avoid co-linearity phenomenon, as this variable is part of the original SAPS 3. Prognostic values were assigned to each variable in the same way than in SAPS II-CNIV (Table 6).

AUC for SAPS II-CNIV and SAPS 3-CNIV were 0.84 and 0.79 respectively. Both scores showed similar calibration performance based on the Hosmer-Lemeshow goodness-of-fit test: (χ^2^ = 5.62, p = 0.69) for SAPS II and (χ2 = 8.41, p = 0.39) for SAPS 3. The bootstrap adjusted AUC for SAPS II-CNIV was 0.83 (95% CI 0.78–0.89) and 0.78 (95% CI 0.72–0.84) for SAPS 3-CNIV.

**Fig 1:**
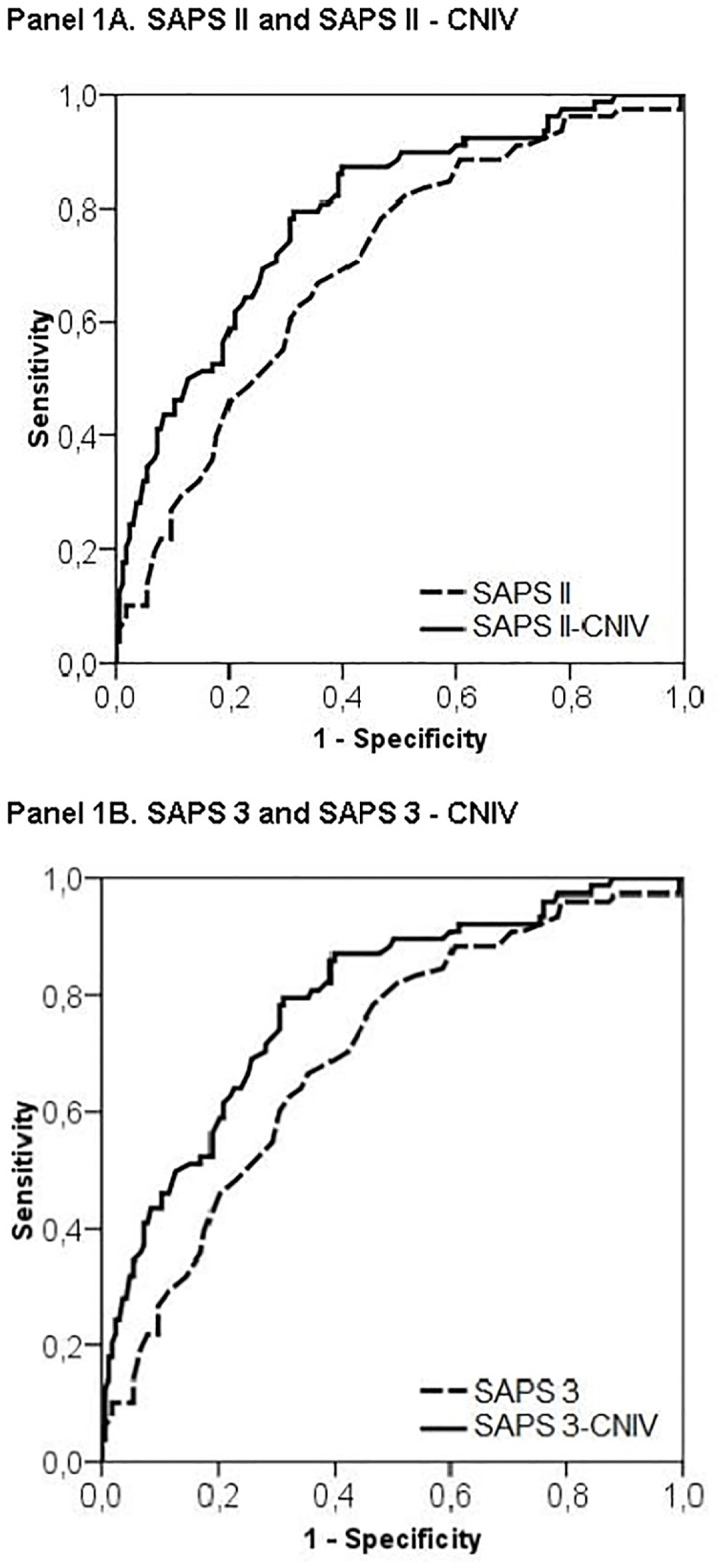
Receiver Operating Characteristic curves in the different prognostic models. (A) SAPS II and SAPS II-CNIV. (B) SAPS 3 and SAPS 3-CNIV.

Table 3 show these values for an easy comparison between primary and customized scores. Fig 1 shows the AUROC curves of primary scores compared to customized versions (Fig 1 Panel A and B).

In an effort to simplify the application of the customized scores, sensitivity analysis was performed on SAPS II-CNIV and 3-CNIV to find critical values which could classify patients in statistically different mortality groups. After this procedure, SAPS II-CNIV was divided in 4 mortality groups and SAPS 3-CNIV in 3. Cut-off values and death probability for each group are shown in Tables 7 and 8.

**Table 7:** Clinical application of SAPS II-CNIV: Death probability according to customized scores.

SAPS II-CNIV (points)	Group 1 (<15)	Group 2 (16–34)	Group 3 (35–46)	Group 4 (>47)
**Number of patients**	46	63	47	75
**Observed mortality (%)**	2.17%	15.87%	36.17%	66.67%

**Table 8:** Clinical application of SAPS 3-CNIV: Death probability according to customized scores.

SAPS 3-CNIV (points)	Group 1 (<34)	Group 2 (34–69)	Group 3 (>69)
**Number of patients**	95	74	72
**Observed mortality (%)**	10.5%	36.49%	56.94%

## Discussion

The results of the current study highlight the importance of customizing SAPS II and 3 with patient-risk factors to improve mortality prediction in patients undergoing NIV in Intermediate Care.

To our knowledge, no previous studies described a complete evaluation of the discrimination and calibration power of SAPS II and 3 in non-invasively ventilated patients. In this study, SAPS II and 3 showed decreased prediction accuracy in terms of discrimination and calibration as compared to original cohort results of both scores in critically ill patients [15,29] and also in ImCU population [30–32].

This fact could be explained by the importance of patient-risk factors in the NIV mortality prediction. Mortality in ARF is highly dependent on respiratory variables at the beginning of the technique [5]. In this context pO2, pCO2, lactic acid and hemoglobin levels at admission have demonstrated to influence mortality prediction in this subgroup of patients [20–24].

Differences in mortality depending on ARF etiology have been extensively described [33–35]. The results in the etiology subgroups of our study are in agreement with previous descriptions; being lower in COPD exacerbation, neuromuscular disease and APE (10–20%), and higher in hypoxemic respiratory failure (50–80%) [5,36,37]. Nevertheless, neither ARF etiology nor previously described physiological variables are included in the original SAPS models.

For this reason, an evaluation combining the use of severity scores and patient-risk factors, could improve the accuracy of mortality prediction in NIV users. In our study, the two customized SAPS II and 3 models appeared to be a synthesis of both concepts, with an acceptable discrimination and calibration power. Additionally, internal validation was assessed by bootstrapping method.

The higher overall mortality of our cohort, in comparison to previously published series [16,36], could be due to differences in case-mix population. The contribution of advanced oncologic (24.5%) and DNR-order patients (29.9%), of our cohort could, at least, partially explain these results. Furthermore, the results of this study support previous descriptions regarding the efficacy of Intermediate Care for NIV application.

Previous studies described the potential of Intermediate Care in terms of cost-containment, rational ICU utilization and triage flexibility for acute patients [38,39]. Recently, Capuzzo et al. [40] described the positive impact of the presence of an ImCU in terms of adjusted hospital mortality for patients admitted in intensive care. Nonetheless, we need more, larger and controlled trials to definitively establish the indications and limitations of the ImCU.

The patients with higher scores and significant mortality risk should be followed carefully, in order to avoid unnecessary delays in transfer to ICU or intubation. However, we need external validation studies to establish the cut-off points for ICU admission.

The most relevant studies for mortality prediction in critical care were multicentric and with extensive number of patients [15, 29]. However, the number of patients in other NIV studies is similar to the present sample [1–4,11–14,36,37]. The cohort of Metnitz et al. [16] derived from a multicenter multinational study of the SAPS 3 database, included 157 patients who underwent PSV. The study was descriptive, without information regarding the calibration and discrimination power of the score and with an observed to expected mortality ratio of 0.79, compared with our SMR of 0.69. These differences could be explained by the overfitting bias of the SAPS 3 score when the validation case mix cohort is substantially different from the original study. These limitations emphasize the need to find specific scores for mortality prediction in NIV. We need more studies, with validation cohorts of the SAPS II-CNIV and SAPS 3-CNIV scores, before its routine application in intensive care.

## Conclusions

SAPS II and 3 models, combined with complementary patient-risk factors, could improve mortality prediction in patients undergoing NIV in the ImCU.

## Abbreviations

ALI: Acute Lung Injury
APACHE: Acute Physiology and Chronic Health Evaluation
ARDS: Acute Respiratory Distress Syndrome
ARF: Acute Respiratory Failure
APE: Acute Pulmonary Edema
AUC: Area Under Receiver Operating Characteristic Curve
CNIV: Customized Non-Invasive Ventilation
COPD: Chronic obstructive pulmonary disease
ICU: Intensive Care Units
ImCU: Intermediate Care Units
NIV: Non-Invasive Ventilation
PSV: Pressure Support Ventilation
ROC: Receiver Operating Characteristic
SAPS: Simplified Acute Physiology Score
SMR: Standardized Mortality Ratio
